# Emergence of leadership in a robotic fish group under diverging individual personality traits

**DOI:** 10.1098/rsos.161015

**Published:** 2017-05-24

**Authors:** Chen Wang, Xiaojie Chen, Guangming Xie, Ming Cao

**Affiliations:** 1 The State Key Laboratory of Turbulence and Complex Systems, Center for Systems and Control, College of Engineering, Peking University, 100871 Beijing, People’s Republic of China; 2 Faculty of Science and Engineering, University of Groningen, Nijenborgh 4, 9747 AG Groningen, The Netherlands; 3 Institute of Ocean Research, Peking University, 100871 Beijing, People’s Republic of China; 4 School of Mathematical Sciences, University of Electronic Science and Technology of China, 611731 Chengdu, People’s Republic of China

**Keywords:** autonomous robots, evolutionary games, robotic fish, group task, personality traits, leadership

## Abstract

Variations of individual’s personality traits have been identified before as one of the possible mechanisms for the emergence of leadership in an interactive collective, which may lead to benefits for the group as a whole. Complementing the large number of existing literatures on using simulation models to study leadership, we use biomimetic robotic fish to gain insight into how the fish’s behaviours evolve under the influence of the physical hydrodynamics. In particular, we focus in this paper on understanding how robotic fish’s personality traits affect the emergence of an effective leading fish in repeated robotic foraging tasks when the robotic fish’s strategies, to push or not to push the obstacle in its foraging path, are updated over time following an evolutionary game set-up. We further show that the robotic fish’s personality traits diverge when the group carries out difficult foraging tasks in our experiments, and self-organization takes place to help the group to adapt to the level of difficulties of the tasks without inter-individual communication.

## 1. Introduction

Collective behaviour in social groups has attracted attention from researchers in different fields, since people have observed that many animal groups and multi-agent systems, in general, can benefit from it [1,2]. For social animals, although individuals may care only about their own well-being and have limited sensing and communication means [3,4], a collective can exhibit flexible, astonishing capabilities in solving a wide range of group tasks, such as foraging, predator avoidance and group cruising [5–7]. For emerging applications of autonomous robots working in an unknown environment, robots have to learn to decide for themselves whether to cooperate with others. The reason is that no engineer can always predict precisely and design with confidence when and how an autonomous robot should sacrifice its own well-being, e.g. battery life and computational resources, to assist others in unknown working situations. In this context, a variety of robotic control strategies have been effectively developed using inspiration from social animals to accomplish team tasks, such as environmental monitoring [8], surveillance [9], exploration [10], pursuit and evasion [11], search and rescue [12], transportation [13] and maintenance in harsh environments [14,15]. However, the performance of such biology-inspired robotic algorithms is sometimes compromised due to those open issues for understanding cooperation mechanisms in social groups in nature, the most demanding among which is why and how group-level cooperation can be achieved through selfish individuals’ local interactions [2,16].

One possible mechanism to induce cooperation in processes of group actions is the establishment of leader–follower relationships between the group members [2,16–18]. In fact, leadership in social groups can exhibit in different forms and thus has inspired a number of seminal studies [19,20]. One influential work by Krause *et al.* [21] on group motion has defined leadership to be the ‘initiation of new directions of locomotion by one or more individuals which are then followed by other group members’. To further avoid possible confusion between the initiation of a group movement and the emergence of leadership to recruit effectively followers, Petit & Bon [18] suggest using the term ‘initiator’ instead of ‘leader’ when specific individuals initiate a collective movement.

Several biological studies have focused on what makes an individual emerge as an initiator among peers [19]. Inter-individual variation, such as body size, age, sex, hunger and risk-taking, may contribute [22–24]; for example, a hungrier individual is more likely to initiate a collective movement to forage. Differences in group members’ preferred courses of action may also contribute [25–27]; in Johnstone & Manica [27], group members each favour a different course of action, which leads to conflicts with each other, and each individual thus has to choose among several options, such as different foraging sites or routes of travel. Since an individual benefits the most from taking its own preferred action besides keeping close to the rest, its movement is a compromise between following more companions (i.e. a good follower) and fulfilling its own preferred action (i.e. a strong initiator). More recently, a number of studies indicate that individuals’ personality traits offer yet another mechanism to stimulate initiators [1,28–31]. Here animal personality is broadly described as the phenomenon of consistent behavioural differences between individuals over time and/or across situations [32]. As one of the best understood personality traits [33], boldness has been investigated in a number of related research studies on leadership [34–36]; the experimental observation of pairs of real fish implies that bolder fish are more likely to initiate a collective movement while shyer fish prefer to follow an initiator [28,29].

Motivated by the above biological studies, we as roboticists thereby investigate the emergence of leadership in a team of robotic fish with diversified behavioural traits. By adopting the same meaning of boldness as in [28,29], we refer a robot’s personality trait, boldness, to its willingness to initiate a collective action, which may adapt as its experience becomes richer. Moreover, the willingness of a robot to follow others’ action is assumed to be inversely correlated to its willingness to initiate. Such specifications on boldness fit the biological observations shown in [28,29]. Then, we are enabled to focus on studying how leadership emerges in a group of interacting heterogeneous robots while all of them take part in a group task that requires each robot to learn to play its role as an initiator, follower or free-rider as it adapts to the success or failure of the group as a whole. We choose a foraging task to be the group task, in which a group of robots has to overcome adversity to reach a source of energy, e.g. recharging station. The adversity they confront is realized by a barrier with unknown and changing weights blocking the way to the energy source, which requires one or more robots in the group to make efforts to contribute cooperatively. So a heavier barrier requires more robots to cooperate, and fewer contributing robots may fail the task. Free-rider robots are likely to exist, who do not contribute but profit by getting access to the energy source once the group successfully removes the barrier. The very fact that the weight of the barrier is unknown to the robots and may change over time makes the foraging task challenging. Note that we intentionally include only one energy source in the set-up and thus robots do not need to resolve the conflict from multiple foraging sites [25–27], so the single dilemma of free-riders dominates and the influence of robots’ behavioural traits on the emergence of group leadership becomes easier to identify.

The robots’ personality traits evolve over time, and are dictated by the updating rules rooted in evolutionary game theory. Before giving more detail about our evolutionary model, we first distinguish our methodologies from the usual simulation-based or animal-test approaches. Simulation studies on emergence of leadership in collective movement usually use evolutionary games to model the evolving population dynamics of life-form agents [19,22,27,30]. When the focus is to draw analytical conclusions based on mathematically tractable derivation, simplifying and sometimes unrealistic assumptions have to be made. Even when mathematical details are of less interest and the freedom for making assumptions is rather ample, some critical features of the physical world, where those simulated evolutionary processes take place, have to be omitted simply because certain dynamical processes [25,37], e.g. hydrodynamics for underwater movements, are too complicated to incorporate. However, hydrodynamics is known to have significant effect on the locomotion and the related behaviours of the fish group [38]. For example, in the context of cooperative group tasks studied in this paper, the hydrodynamics around the fish group may greatly affect the outcomes of the robots’ pushing behaviours (i.e. the success or failure in the tasks) [39]. Experimental study with real animals mimics the real-world scenarios in controlled laboratory environment, but always faces constraints on available tools that can be applied to animals, such as non-intrusive sensing and measurement [26,28,29,40]. In sharp comparison, we develop a new framework using a multi-robotic-fish system as the experimental tool. Since the design of the robotic fish and its locomotion control imitates the physical features of real fish, which have been commonly ignored in most of the simulation studies, the testbed provides new perspectives to examine collective motion. In particular, for the schooling fish foraging tasks, the fish’s energy consumption is closely related to the hydrodynamics in the foraging environment [41,42]; however, up to now there are no appropriate theoretical or simulation models for fish’s energy costs dealing with the local currents in nature or induced by their fellow fish’s movement. So the biomimetic robotic fish, generating and dealing with hydrodynamics as they carry out the foraging task, provide an opportunity to gain insight into how evolution at the behaviour level takes place interacting with the complicated dynamical changes in the surrounding physical world. Another advantage of using robotic fish is that there is the potential to extend the robotic fish system to a heterogeneous system consisting of both robotic and real fish, which allows us to steer the behaviour of the robotic fish to influence the overall fish group. Such a heterogeneous system may provide us a new way of understanding fish behaviours in nature, whereas simulations may be difficult to carry out for such mixed populations of real and robotic fish. Moreover, in this paper, we propose a game model for the personality trait updating rule which takes its extensive form (or tree form), while most of the existing studies use game models in their strategic form (or normal form) [22,27,30]. The extensive form can be more flexible to describe decision-making processes in stages and thus closer to reality.

In what follows, to investigate the emergence of leadership in foraging tasks for robots with heterogeneous personality traits, we develop a new framework that uses the multi-robotic-fish system as the experimental tool in which robots’ personality traits adapt over time. We model the dynamic process of foraging by a modified *N*-player snowdrift game in its extensive form, since snowdrift games are typically used to study contributing versus free-riding tendencies (i.e. the dilemma of free-riders) in multi-player games. In each round of the game, each robotic fish has to choose whether to initiate a collective action (i.e. to be an initiator). Once an initiator appears, other robots choose to become a follower taking part in overcoming adversity or a free-rider. The pay-off of each robot depends on both whether the group task has been finished successfully and whether the robot itself has made efforts. We define the personality trait, boldness, of each robot to be its strategy in this repeated game that updates under a self-learning rule after each round of the game. The robots with high values of the personality trait (i.e. bolder fish) are more likely to initiate a collective movement, whereas those with low values (i.e. shyer fish) prefer to follow an initiator; once another robot has become an initiator, the bolder fish are less likely to contribute to the group task (i.e. less responsive), and consequently are more likely to become a free-rider. This assumption fits with the biological observation [28,43,44]. The effective leadership [27] is then defined to show how a robot influences others in the group. We say effective leadership has emerged in the group when the foraging task is indeed accomplished by an initiator with one or more following robots [20]. We will show through experiments that the personality traits in a group diverge more as the difficulty of the group task increases, which is critical to guarantee the successful accomplishment of the tasks. Moreover, we find that the robotic fish group has the ability to self-organize in order to adapt to the difficulty of the tasks, even though the difficulty may change over time and is unknown to the fish group in which no fish can communicate with each other. Since the testbed keeps a number of realistic representations of the real world and the behaviour of the biomimetic robotic fish evolves according to evolutionary principles, the obtained observation may help researchers to understand better the evolution of leadership and emergence of cooperation in real fish schools; more importantly, the evolutionary approach for leadership may inspire new protocols to coordinate autonomous robots.

## 2. Material and methods

### 2.1. Model for evolving personality traits and emergence of leadership

As indicated by biological studies [1,28–30], differences in personality traits offer an opportunity to generate group leadership that is the key to start and accomplish collective action. This inspires us to introduce *a personality trait, boldness*, to the social agents, which is defined by the willingness of an agent to initiate a collective action (i.e. to be an *initiator*) in performing the foraging task. The same intention, as discussed in [1,29,30,45], is inversely correlated to the willingness to be a *follower* (to follow some initiator). More specifically, an agent with a bolder personality trait has a higher probability of being an initiator and lower probability of being a follower, while an agent with a shyer personality trait has a lower probability of being an initiator and higher probability of being a follower. When an initiator indeed has followers, we also call it a *leader*. Obviously, both the initiator and its followers (if any) in the group cooperate to overcome adversity in order to complete the foraging task. Note that an agent may, and probably will, choose to wait for the other agents to overcome adversity, in which case it becomes a *free-rider*. When the foraging task is indeed accomplished by an initiator with one or more followers, we say *effective leadership* has emerged in the group [20].

Personality traits and thus the roles of initiator, follower or free-rider of the agents may vary and evolve. We model this dynamic process by a modified *N*-player snowdrift game in its extensive form, since snowdrift games are typically used to study contributing versus free-riding tendencies in multi-player games. For each agent *i* (*i*=1,2,…,*N*) its personality trait, boldness, is denoted by *s*_*i*_∈(0,1). For convenience, an agent *i* is said to be ‘bold’ if *s*_*i*_>0.7 and ‘shy’ if *s*_*i*_<0.3. We use *n*_*B*_ to denote the number of bold agents in the group. To reflect the biological observation that a bold agent is more likely to become an initiator [28], we associate each agent with an exponentially distributed random variable $t_{i}^{D}$, called ‘decision time’, whose mean (expected value) is 1/*s*_*i*_; an agent will take the initiative to lead the group task if, when its decision time comes, no other agent has become the initiator yet. Thus, the agent whose decision time is the shortest becomes an initiator. More specifically, the sequence of $t_{1}^{D},t_{2}^{D},\ldots ,t_{N}^{D}$ is independent and identically distributed (i.i.d) according to the exponential distribution with the rate parameter *s*_*i*_ whose probability density function is written as

2.1 $$f(t_{i}^{D})=\left\{ \begin{array}{ll} s_{i}\;e^{-s_{i}t_{i}^{D}} & t_{i}^{D}\geq 0\;(s_{i}>0)\\ 0 & t_{i}^{D}<0 \end{array}\right.,\;i=1,2,\ldots ,N.$$

One can obtain that the index of the variable which achieves the minimum is distributed according to

2.2 $$\mathrm{Prob}\left(t_{m}^{D}=\min\left\{t_{1}^{D},t_{2}^{D},\ldots ,t_{N}^{D}\right\}\right)=\frac{s_{m}}{s_{1}+s_{2}+\cdots +s_{N}},\;m=1,2,\ldots ,N.$$

In other words, the probability of an agent becoming an initiator is proportional to its personality trait *s*_*i*_, which fits well with the biological observation that a bold agent is more likely to become an initiator. Note that since no agent knows about the $t_{i}^{D}$ of other agents, each agent does not know *a priori* whether itself might be an initiator or leader in the group. Once an initiator appears, with the probability $1-s_{i}^{2}$ each of the other *N*−1 agents becomes a follower participating in overcoming adversity, and consequently each of them becomes a free-rider with the probability $s_{i}^{2}$. Here we have specified the inverse correlation between boldness and likelihood to become a follower that we described before to be a quadratic function. So we have specified how an agent takes its role of initiator, follower or free-rider.

For each round of the task, which we also refer to as a stage game from now on, since we will formalize the set-up using evolutionary game models, each agent takes the action to cooperate and is thus a *C*-player if it is the initiator or a follower; otherwise when it is a free-rider, it is a defector or *D*-player. We denote the cost that a *C*-player has to endure by *c*>0 for pushing the obstacle. If indeed enough *C* actions in the group lead to the successful overcoming adversity, the benefit that every agent in the group receives is *b*>*c*; otherwise, the benefit for every agent is zero. Hence, the pay-offs of *C*-players and *D*-players, denoted by *p*_*C*_ and *p*_*D*_ respectively, are

2.3 $$\begin{array}{r} p_{C}=rb-c\;\mathrm{and}\;p_{D}=rb, \end{array}$$

where *r*=1 if the group accomplishes the task and *r*=0 otherwise.

After each stage game, the personality of the initiator changes depending on whether the task has been finished successfully. This design reflects the biological observation that experiences of group members influence their tendency to initiate or follow [29]. Let the current index of the stage game be *k* (*k*=0,1,2,…) and the label of the current initiator be *j* (*j*=1,…,*N*); the update rule [46–48] is

2.4 $$s_{j}(k+1)=r\lambda +s_{j}(k)(1-\lambda ),\;k=0,1,2,\ldots ,$$

where *λ*∈(0,1) is the update rate and *r* defined in (2.3) becomes the reinforcement variable. So the update rule (2.4) introduces a positive feedback to the initiator in the sense that an initiator becomes bolder (i.e. *s*_*i*_ getting closer to one) and more likely to be an initiator again in the future if it has just initiated a successfully accomplished task; otherwise, it becomes shyer (i.e. *s*_*i*_ getting closer to zero) and less willing to initiate a collective movement. So the key component in this game set-up is to enable self-learning for the initiator. This is rooted in our motivation to study the relationship between the personality trait, boldness and effective leadership.

### 2.2. Experimental implementation

**Figure 1.**
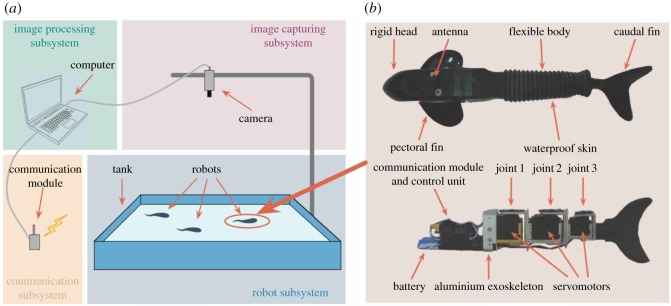
Experimental platform used to conduct our proposed framework to investigate the behaviours of biomimetic robotic fish groups. (*a*) Hardware system of the experimental platform. (*b*) Mechanical configuration of a biomimetic robotic fish. The upper is the top view after encapsulation. The lower is the side view of the interior mechanical structure. (Figure drawn by Chen Wang; photograph taken by Chen Wang, Peking University.)

To implement the proposed model, we use the multi-robotic fish testbed as the experimental tool. The experimental platform that collects real-time pose information (position, direction and speed) of the robot fish is shown in figure 1*a* and electronic supplementary material, figure S1, while the biomimetic robotic fish is shown in figure 1*b* [13,49].

The experimental platform consists of a server computer, an overhead camera, a wireless communication module and a robot subsystem (figure 1*a*). The overhead camera captures images of the tank per 40 ms and then sends them to the server computer, where images are processed to obtain the pose information of the robots. The upper computer can exchange information with robots (i.e. sending pose information to the robot and receiving feedback information from the robot) through the wireless communication module. The software system architecture of the experimental platform consists of four modules (electronic supplementary material, figure S1): GUI (graphics user interface) module, robot module, image capturing and processing module, and communication module. The pose information of the robot is calculated in the image capturing and processing module and displayed together with the captured image in the GUI window in real time. Through the GUI, one can set the parameters of the experiments. Both the parameters and the pose information are sent to the robot by the communication module in real time.

The robotic fish, which measures 40 cm long and weighs 0.85 kg in total, consists of a streamlined head, a flexible body and a caudal fin (figure 1*b*, upper). The head, which is made of fibreglass, accommodates an onboard control unit, a duplex wireless serial-port communication module and a set of rechargeable batteries (figure 1*b*, lower). The battery pack is placed at the bottom of the fish to lower its centre of mass and consequently stabilize the vertical posture. A pair of fixed pectoral fins is rigidly attached to the sides of the head to ensure the roll stability when the robotic fish swims. Accordingly, the robotic fish’s locomotion is confined roughly to a horizontal two-dimensional space. The body of the robotic fish contains three revolute joints that are linked together by aluminium exoskeletons. Each joint is driven by a R/C servomotor, which controls its relative joint angle with respect to those of its adjacent joints. The caudal fin is attached to the third joint. The tailor-made waterproof rubber skin covers the whole flexible part of the body from the neck to the tail. The density of the robotic fish is just a little bit less than that of the water so that it swims just below the water surface to keep the antenna above the water.

All the onboard electronic devices of the robotic fish are powered by four 5 V rechargeable Ni–MH batteries. The onboard control unit, which is based on a micro-controller, Atmel ATmega 128, runs control algorithms and generates the control commands in the form of pulse width modulation (PWM) to drive the three R/C servomotors at the three joints in real time. The servomotors at the first two joints are Futaba S3003 with a maximum recommended torque of 3.2 kg cm and a working speed of 0.23 s/60° at 4.8 V. The smaller servomotor of the third joint is Futaba S3102 with a maximum recommended torque of 3.7 kg cm and a working speed of 0.25 s/60° at 4.8 V. The real-time communication with server computer and other robotic fish can be achieved through the communication module WAP300C. We refer to [13,49,50] for readers who are interested in more technical details about the robotic fish and its locomotion control, and the experimental platform.

Note that the experimental platform as well as the robotic fish is designed as a general testbed so that it can meet a variety of requirements for different research purposes. In this study, to make the testbed as a decentralized system in which the robotic fish independently takes part in the group task without any centralized controller or inter-individual communication, we add the following three constraints to the above general testbed. First, the only information sent from the server computer to each fish is the robots’ pose information. Second, the proposed model in §(a) is independently executed on the onboard control unit inside each fish. Third, the fish are not allowed to communicate with each other.

### 2.3. Foraging scenario

**Figure 2.**
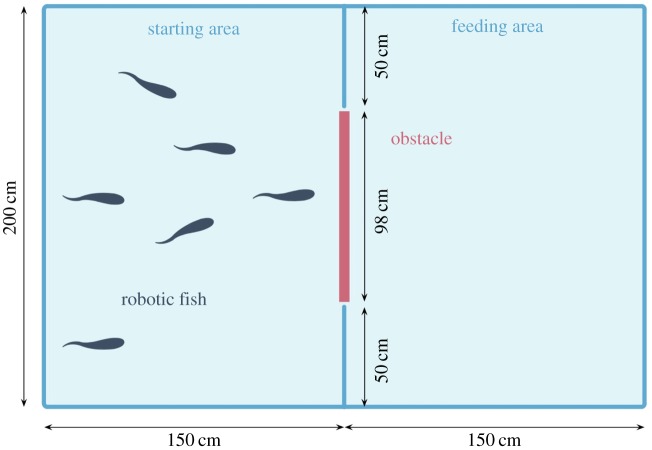
Schematic view of the experimental set-up for the foraging tasks. The tank is divided into two areas, the starting area on the left and the feeding area on the right, which are separated by a fixed screen with a removable obstacle in the middle. The robotic fish group has to swim from the starting area to the feeding area, and this task can only be accomplished after the group removes the obstacle blocking its way. (Figure drawn by Chen Wang.)

Based on the above experimental implementation, the foraging task is specified as a foraging scenario in which a group of *N*>1 robotic fish needs to remove an obstacle to complete the foraging task. The schematic view of the experimental set-up is shown in figure 2. The tank (300×200×40 cm, length × width × depth) is divided into two areas, the starting area on the left and the feeding area on the right, which are separated by a fixed screen with a removable obstacle (98×8×26 cm, length × width × height) in the middle. The robotic fish group has to swim from the starting area to the feeding area, and this task can only be accomplished after the group removes the obstacle blocking its way. Four obstacles with different weights are used to implement four difficulty levels of the foraging tasks. We label the obstacles by *O*_1_, *O*_2_, *O*_3_ and *O*_4_ according to the ascending order of their weights; more concretely, the weights of *O*_1_, *O*_2_, *O*_3_ and *O*_4_ are 1.6 kg, 2.0 kg, 2.5 kg and 2.9 kg, respectively. Given the mechanical structure of the fish, the way that a robotic fish tries to move an obstacle is to push its head against the obstacle and swim forward at the same time. The fish are identical in their design and production, and their capabilities for pushing the obstacle are thus the same. In fact, each fish has only limited pushing power and the obstacle can be removed only if several fish cooperate together. Obviously, heavier obstacles require more fish.

### 2.4. Experimental settings

**Figure 3.**
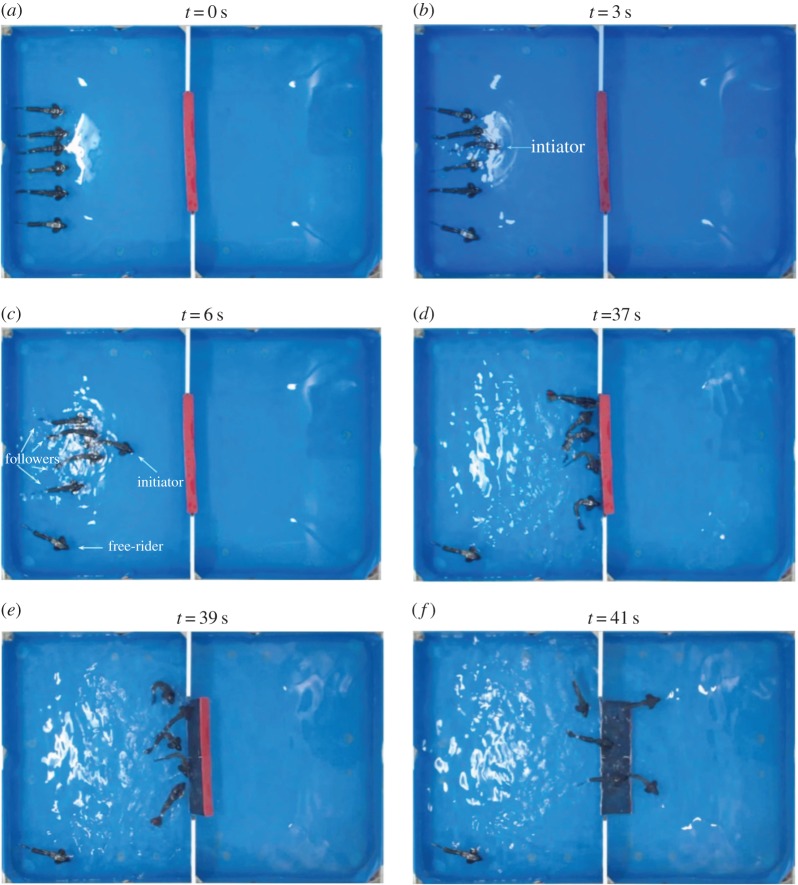
Snapshots of the foraging tasks implied by our experimental platform. A group of six biomimetic robotic fish starts a foraging task at *t*=0 s (*a*). At around *t*=3 s, one initiator emerges (*b*). Then some other four fish become followers joining the initiator to remove the obstacle, while one fish in the group choose to be a free-rider (*c*–*e*). At *t*=41 s, the fish group accomplishes the foraging task successfully. (Photograph taken by Chen Wang, Peking University.)

Owing to hardware constraints, such as the battery life, of the real robotic fish system, we choose the small group size *N*=6 for experiments. The update rate *λ* varies from 0.4 to 0.9 with the step size 0.1 so that the total number of tasks within a single evolution process is not too large. In fact, we consider that each evolution consists of 200 tasks. To guarantee the implementation of large numbers of experiments, we also require that a task has to be finished within 60 s and so no matter how many fish are participating in the pushing motion, if the execution has passed 60s, the group fails. We set *b*=2 and *c*=1 to fulfil the requirement of *b*>*c* for the snowdrift game and initialize *s*_*i*_ between 0 and 1 for each fish *i* randomly and independently. Figure 3 shows the snapshots of one experiment in which five collaborating fish remove the obstacle successfully; more concretely, in a six-fish team, one initiator emerges and drives some other four to cooperate to remove the obstacle, and the team successfully accomplishes the foraging task.

### 2.5. Data analysis

The aim of our study is to investigate how personality traits evolve and effective leadership emerges in the group of robotic fish when doing foraging tasks. Thus, we focus on the evolving strategies *s*_*i*_ representing the personality traits and further the roles of robots. We also focus on the performance of the fish groups. Considering the evolving roles of robots, let *n*_*C*_ denote the number of cooperating fish in the group. While, as we mentioned, *n*_*B*_ denotes the number of bold fish whose boldness satisfies *s*_*i*_>0.7. In order to investigate the shift of the key role of an initiator, we use *I*_*n*_ to denote the label of the initiator in each task. We further use *p*_*i*_ to denote each fish *i*’s percentage of being an initiator in the three roles (i.e. initiator, follower and free-rider) during a single evolution. Then the standard deviation (s.d.) of *p*_*i*_ of all *N* robotic fish is denoted by

2.5 $$d_{I}=\sqrt{\frac{1}{N}\sum_{i=1}^{N}{\left(p_{i}-\frac{\sum_{i=1}^{N}p_{i}}{N}\right)}^{2}},$$

which indicates directly the degree of diversity of the personality traits during a single evolution. In other words, the degree of diversity *d*_*I*_ is a measure summarizing the distribution of the likelihood of being an initiator over all robotic fish in the group during a single evolution. To study the performance of a fish group when doing foraging tasks, the success rate is considered to be the most important index. We also record the decision time, the smallest $t_{i}^{D}$ of all group members (i.e. the $t_{i}^{D}$ of the initiator), used for each task. The average cost and average pay-off of the fish group are also shown. In order to get the statistical results for a single evolution, the average value of *s*_*i*_, *n*_*B*_, *n*_*C*_, *d*_*I*_, $t_{i}^{D},$ average cost, and average pay-off are all calculated using the last 100 tasks in the total 200 tasks to characterize a more stable situation of the evolutionary process.

### 2.6. Quantify the difficulty of a foraging task

The difficulty of the foraging task can be naturally quantified by the weights of obstacles. However, it is intuitive to use the number of cooperating fish needed to work on the task for quantifying the difficulty. Because of the unavoidable variations in how a robotic fish executes its pushing motion, for the same obstacle, different numbers of cooperating fish are needed in different realizations of the experiment. We list in electronic supplementary material, figure S2, the experimental results on the relationship between the number *n*_*C*_ of cooperating fish and the success rate of the group tasks for different obstacles, where each cell of the table corresponds to the average over 100 realizations.

One can conclude in general that the difficulty levels increases from *O*_1_ to *O*_4_ and the success rate is sensitive to the number *n*_*C*_ of cooperating fish in different ranges. More precisely, when *n*_*C*_=1, the fish player only succeeds in 17% of tasks for obstacle *O*_1_ and it is impossible to remove the other three obstacles. When *n*_*C*_ increases to 2, players always accomplish the task for *O*_1_ while still fail for the other three obstacles. When *n*_*C*_ increases to 6, the players are always able to remove the obstacles. Note that there is a chance for potential free-riders no matter which obstacle is present, so the appearance of effective leadership does not require all the fish to become cooperative.

### 2.7. Reference model without introducing the personality trait

In comparison with the proposed model, we consider a reference model without the personality trait for the robotic fish system. We assume that the strategy ${\hat{s}}_{i}\in (0,1)$ for fish *i* simply represents the probability of fish *i* to participate in pushing the obstacle. Thus, in each stage game, fish *i* chooses to be either a *C*-player or *D*-player with the probability of ${\hat{s}}_{i}$ or $1-{\hat{s}}_{i}$, respectively. We consider two cases, without and with evolution, respectively. In the case without evolution, the strategy of each fish is randomly chosen and stays the same during the evolutionary process. In the case with evolution, the initial strategy of each fish is randomly chosen, and after each task, every *C*-player’s strategy updates according to the standard update rule (2.4). Other experimental settings are the same as those used in our main model with the personality trait. We find that the strategy update rate rarely influences the evolutionary outcome. Thus we set *λ*=0.9 in the case with evolution.

All notations and variables, which appear in the following sections, are listed in table 1 with short descriptions and their locations.

**Table 1.** List of notations and variables.

	notations	explanation	
each fish *i*	*s*_*i*_	personality trait (boldness) of fish *i*	§2.1
	$t_{i}^{D}$	decision time of fish *i*	
update rule	*r*	reinforcement variable	equation (2.4)
	*λ*	update rate	
a single foraging task	*O*_1_,*O*_2_,*O*_3_,*O*_4_	difficulty level of the foraging task	§2.3
	*p*_*C*_	pay-off of each *C*-player	equation (2.3)
	*p*_*D*_	pay-off of each *D*-player	
	*I*_*n*_	label of the initiator	§2.5
	*n*_*B*_	number of bold fish	
	*n*_*C*_	number of cooperating fish	
a single evolution	*p*_*i*_	fish *i*’s percentage of being an initiator	
	*d*_*I*_	degree of diversity in a group	

## 3. Results

### 3.1. Evolution confronting tasks with fixed difficulties

To investigate the evolution of personality traits and emergence of leadership, we first conduct the experiments repeatedly with four fixed tasks of distinct difficulty levels.

**Figure 4.**
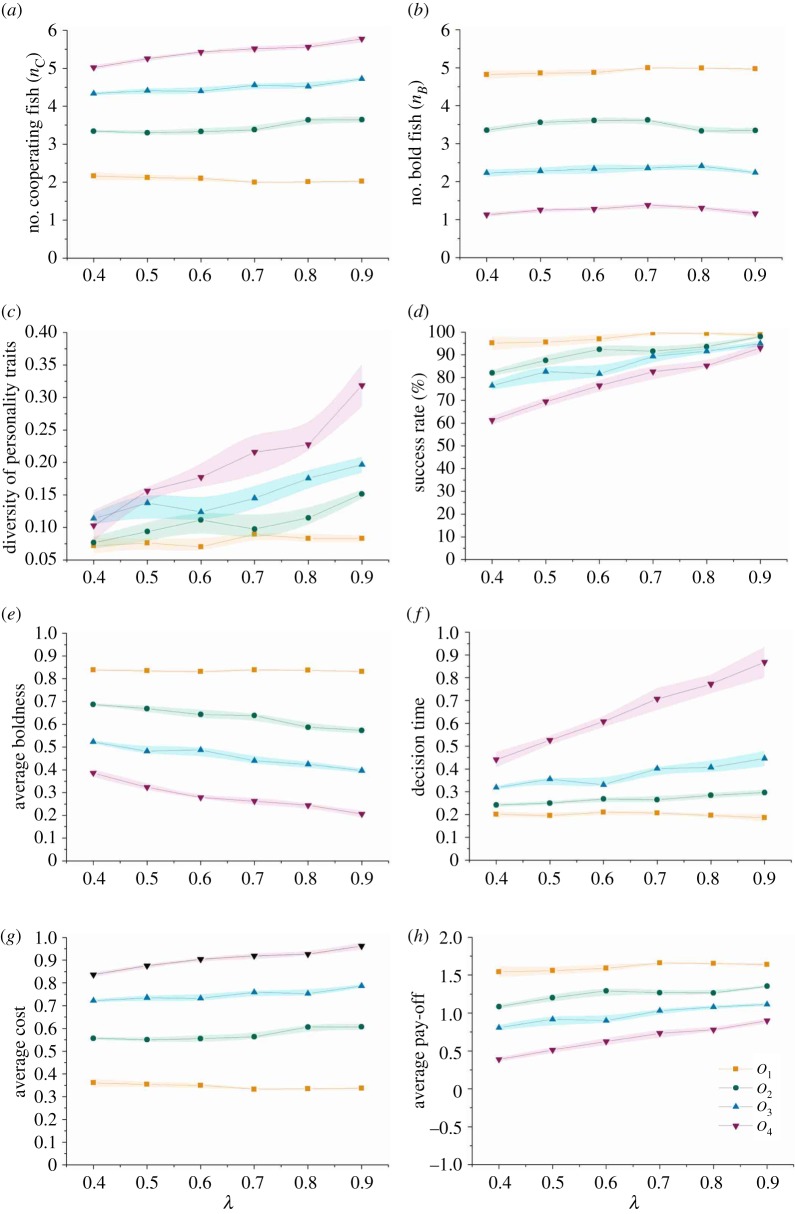
Statistical results of the evolution with fixed tasks of different difficulty levels. *O*_1_, *O*_2_, *O*_3_, *O*_4_ denote the difficulty levels of the foraging task which increase from 1 to 4. Each data point is the average of five runs and the standard error (shaded area) is shown as well. Note that the variations are expected to be small since each of the five runs itself is the average of 100 trials. (*a*,*b*) The number of cooperating fish (i.e. *C*-players) and bold fish, respectively; (*c*) the diversity of personality traits; (*d*) the success rate; (*e*) the average personality traits, i.e. average boldness; (*f*) the decision time of the fish group and (*g*,*h*) the average cost and average pay-off, respectively. (Figure drawn by Chen Wang.)

We first focus on the statistical results in figure 4. From figure 4*a*, one can see that the number of cooperating fish (*n*_*C*_) is smaller when the task is easier, and in comparison *n*_*C*_ is bigger when the task is more difficult. The very fact that more fish in the group participate in pushing the obstacle when the task is more difficult implies that, although there is no communication or sharing of information between the fish, the personality-based evolution enables the fish group to respond adaptively to different difficulty levels of the tasks. On the other hand, in figure 4*b* it is clear that the number of bold fish (*n*_*B*_) decreases as the difficulty of the tasks increases. This means that fewer fish are willing to take the role of initiator when facing more difficult tasks. In figure 4*c*, one can observe that the degree of diversity of the personality traits (*d*_*I*_) increases as the strategy update rate (*λ*) increases and the same trend holds for the corresponding success rate as shown in figure 4*d*. Although the success rate decreases as the difficulty of the task increases, it still keeps at a higher level and gets close to 100% as the update rate (*λ*) increases. Furthermore, in figure 4*e*,*f*, as the difficulty of the task increases, the average boldness of the *N* players decreases and then the decision time of the group increases. This implies that the group takes a longer time to have an initiator when the task is more difficult. In addition, from figure 4*g*,*h*, the average cost of the group increases as the difficulty of the task increases, while the average pay-off of the group decreases. Moreover, we want to emphasize that the strategy update rate (*λ*) is always important for the group’s performance, although its effect, especially for the case when facing the easiest task, cannot be easily identified in the statistical results in figure 4. We will discuss this in the following §(b).

To gain insight into the evolutionary process, we choose two typical processes when the strategy update rate is set to be small (*λ*=0.4) and large (*λ*=0.9), respectively, for the most difficult level of the foraging task (the task with obstacle *O*_4_). The results are shown in electronic supplementary material, figure S3. For both processes, one can check from the figures that the number of cooperating fish (*n*_*C*_) is around 5 or 6 under which the task can be completed almost for sure, while the personality traits (*s*_*i*_) diverge. Now we compare the two typical evolutionary processes. When the strategy update rate is large (*λ*=0.9), the degree of the diversity is 0.37 and the success rate is 96%. The personality traits of the individuals in the group show a strong tendency for diversity, which can be seen from the dynamics of the personality traits (*s*_*i*_) in the figures. As a consequence, the process of role differentiation has occurred after a few steps of the evolution, which is clearly indicated by the label of the initiator (*I*_*n*_) in the figures. Specifically, the curve of *I*_*n*_ shows that an initiator emerges in the group and remains to be an initiator for a period of time. Note that role-switch also takes place in the group when such a relatively fixed initiator is replaced by some other group members. A histogram is used to show the percentage of different roles for each fish during a single evolution. When the strategy update rate is small (*λ*=0.4), the degree of the diversity is 0.06 and the success rate is only 56%. In this case, the diversity of personality traits is not obvious and the percentage of the roles for each fish is similar.

### 3.2. Evolution confronting changing tasks

**Figure 5.**
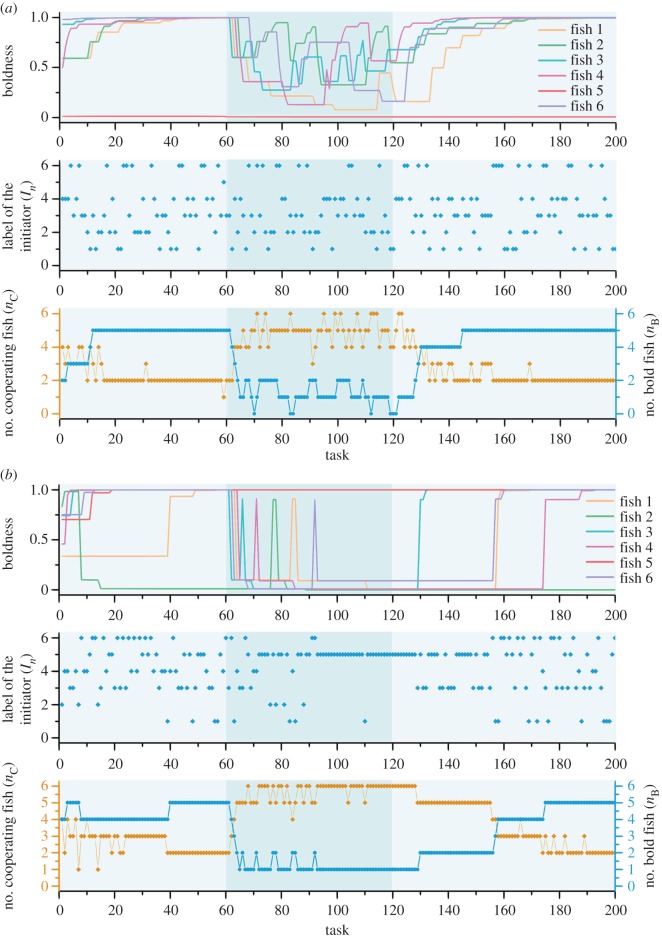
A single evolution with changing tasks. The light shadow shows the period of facing the easiest task with difficulty level 1 while the dark shadow shows the period of facing the most difficult task with difficulty level 4. (*a*,*b*) The personality trait, i.e. boldness, (*s*_*i*_) of each fish, the label of the initiator (*I*_*n*_), the number of cooperating fish (i.e. *C*-players) (*n*_*C*_) and the number of bold fish (*n*_*B*_) are shown in each figure. (*a*) The case when the update rate is small (*λ*=0.4), in which case the success rate is 84%; (*b*) the case when the update rate is large (*λ*=0.9), in which case the success rate is 91.5%. (Figure drawn by Chen Wang.)

Now we look at the situation when different obstacles are present during a single evolution process. We still choose two typical situations of small (*λ*=0.4) and large (*λ*=0.9) strategy update rate. Each evolution consists of 200 tasks, during which the starting period (the first 60 tasks) and the ending period (the last 80 tasks) are set to be easy (with obstacle *O*_1_), while the middle period (task 61–120) is set to be difficult (with obstacle *O*_4_). The results are shown in figure 5.

In figure 5, when the difficulty level of the foraging tasks changes from easy to difficult, both the number of cooperating fish (*n*_*C*_) and the number of bold fish (*n*_*B*_) quickly converge to the level which is suitable for the difficulty. On the other hand, when the difficulty level decreases, an interesting but counterintuitive phenomenon appears, which is that the group can still quickly adapt itself to the new difficulty level under a low update rate (*λ*=0.4), whereas a higher update rate (*λ*=0.9) requires more time for the adaptation. To explain this phenomenon, we first focus on the period of facing the most difficult task indicated by the dark shadow, and then check what happens when the difficulty changes to the easiest level indicated by the light shadow in figure 5. For the case under the low update rate in figure 5*a*, facing the most difficult task, five of six fish in the group have the same level of willingness to be an initiator from a statistical point of view, and they initiate in turns. Owing to the small update rate, in the first few tasks after the difficulty level decreases, the five fish still have the same probability to be an initiator, and the number of cooperating fish remains at a high level of 4–6 which guarantees the task to be successful. Consequently, the five fish have the similar probability to increase their boldness gradually. In other words, the five fish increase (respectively, decrease) their willingness to be an initiator (respectively, a follower) almost in synchrony. As a result, the number of cooperating fish and the number of bold fish quickly converge to the suitable level in accordance with the task’s difficulty. However, the situation is quite different for the case under high update rate in figure 5*b*. When facing the most difficult task, one of the six fish, compared with others, has a very high level of willingness to be an initiator, and it becomes and then almost remains to be an initiator. Owing to its much higher level of willingness than others, it still acts as an initiator for a period of time after the difficulty level decreases, whereas other fish have low probabilities to become an initiator and are thus with low probabilities to increase (respectively, decrease) their willingness to be an initiator (respectively, a follower). It leads to the fact that the number of cooperating fish and the number of bold fish need more time to adapt for the decreasing of the difficulty level.

Figure 5*a* shows another phenomenon under the low update rate in which, although the difficulty level of the task changes, the group exhibits the same performance when five of the six fish act as an initiator in turn. This phenomenon can be explained by the fact, which is reflected in the upper subfigure of figures 4*c* and 5*a*, that the group keeps a lower degree of diversity of personality trait under different task difficulty levels of the tasks with the low update rate. From another perspective, the evolution with changing tasks shown in figure 5 provides the micro phenomena which cannot be easily seen in the statistical results focusing on macro phenomena in figure 4. Concretely, although the statistical results in figure 5 show that the effects of the strategy update rate (*λ*) reduce as the task’s difficulty decreases and even to no effect in the easiest task, the strategy update rate (*λ*) is always important for the group’s performance which can be seen in the upper and bottom subfigures of figure 5.

### 3.3. Importance of personality traits

We verify the important role of personality trait in determining the group’s performance in doing the group tasks by comparing with the reference model without introducing personality traits (for the details of the model and experiments, see §2.7).

Without personality traits (see electronic supplementary material, figure S4), the group performance becomes worse as the difficulty of the task increases, no matter whether the fish’s strategies evolve or not. More specifically, as the difficulty of the task increases, both the success rate and the average pay-off decrease quickly. In the situation when the fish’s strategies evolve, all the group members’ strategies converge to a common value. However, this common value decreases as the difficulty of the task decreases. In other words, more fish participate in pushing the obstacle if the task is easier, while fewer fish do so if the task is more difficult. This leads to the unexpected result that the group makes excessive efforts for easier tasks, while the group lacks cooperating fish for more difficult tasks. That is the crucial reason that the success rate is very low and, further, the group’s performance is worse. In addition, we have compared the group’s performance in the situation without introducing personality traits (electronic supplementary material, figure S4) and that with personality traits (figure 4); it is obvious to see that both the success rate and the average pay-off of the group modelled with personality traits are higher for all difficulty levels of the foraging tasks. We conclude that the fish’s personality traits and their diversity during the evolution result in an improved group performance through self-organization and learning.

## 4. Discussion and conclusion

Leadership is one of the possible mechanisms to induce collective behaviour in nature, which is of benefit to many social groups and multi-agent systems in general [1,2,16–18]. Several biological studies indicate that the emergence of leadership in animal groups correlates with individuals’ personality traits [1,28–31]. In this paper, we focus on one of the best understood personality traits, boldness [33–36], and develop a new framework that uses a multi-robotic-fish system as the experimental tool to investigate the emergence of leadership in foraging tasks for robots with heterogeneous levels of boldness. Our experimental results reveal two features, *self-organization* and *divergence of personality traits*, that characterize the group’s behaviour under a evolutionary game setting.

Self-organization in social groups has been widely investigated [51,52], and several models have gained great success in explaining how self-organization can arise from the interaction of individuals following simple rules [53,54]. Our results imply that the self-organization can also be induced by the variations of individual’s personality traits in the group, particularly the way in which the group members respond to one other according to their boldness. A similar result has been observed in studies of a pair of real fish [28], where the variation among individual group members in the rules that they follow has been shown to enhance the self-organization in fish groups.

In the following two subsections, we discuss more details about the two features, self-organization and divergence of personality traits, of our multi-robotic-fish groups.

### 4.1. Self-organization without inter-individual communication

In view of the evolution of individual personality traits in the group, we identify that more bold fish emerge in the group when the foraging tasks are easier. On the other hand, more fish in the group participate in pushing the obstacle when the task is more difficult. However, note that in our study, the difficulty level of the task is unknown to the fish group and the fish do not communicate with each other. Each fish makes the decision independently and updates its personality trait by self-learning. Thus, the above phenomena imply that the fish group has some ability of self-organization to adapt to the difficulty level of the group tasks. Further evidence of self-organization in the group is found in the experiments when the difficulty level of tasks changes, which shows that the group can still adapt itself to the changing tasks. Moreover, when the difficulty level decreases, an interesting but counterintuitive phenomenon appears, in which the lower update rate can steer the group quickly to adapt itself to the new tasks, whereas a higher update rate requires more time for the adaptation.

Naturally one looks for the key mechanism for the evolving multi-fish systems to have the ability of self-organization. We identify the fish’s personality trait and its evolution to be our answer. Considering the difficult tasks for which a lot of cooperating fish are required to remove the obstacle, the initiator is highly likely to fail the task because of lack of enough followers, and consequently, its willingness to initiate is more likely to decrease because of the feedback by the self-learning update rule (2.4). In comparison, for easy tasks, the initiator is more likely to succeed with its followers because the easy tasks do not need too many cooperating fish. Then its willingness to initiate may increase with a high probability. In summary, more bold fish emerge in the group when the tasks are easier. On the other hand, a bolder fish is unwilling to be a follower. Then for a group with more bold fish, the followers are fewer and thus the cooperating fish are also fewer. In short, more fish in the group participate in pushing the obstacle when the task is more difficult.

### 4.2. Divergence of personality traits

We further study the degree of diversity of the personality traits. It can be confirmed from the experiments that the degree of personality trait increases as the difficulty of the obstacle-removing task increases, and similar effects exist for the larger strategy update rate. Moreover, the process of role differentiation has occurred after a few steps of the evolution. Typically, for larger strategy update rates, an initiator emerges in the group and remains so for a period of time. The phenomenon of role-switching in the group shows up when such a relatively fixed initiator is replaced by some other group members.

The reason that divergence of personality traits emerges for difficult tasks and for large update rates can be explained as follows. As we mentioned before, for difficult tasks, the initiator may fail the task with a high probability and its willingness to initiate is more likely to decrease, i.e. it becomes shyer. However, once a fish becomes an initiator and leads the followers completing the task, its willingness to initiate will increase while many other fish have already ended up with lower willingness to initiate, i.e. become shyer, during the evolution. Thus, the bolder fish is more likely to be an initiator again. Since many other fish are shyer and they are more likely to be followers, the bolder fish succeeds with higher probability so that its personality trait may become even bolder. In comparison, even if a shy fish happens to be an initiator, it may become shyer and shyer with high probability. Then the divergence of personality traits emerges during the evolution. It is natural that both the increasing difficulty of the task and increasing update rate will reinforce the degree of diversity of personality traits, and then further increase the success rate of the group.

One may explore even further when taking the personality trait to be the fish’s confidence as an initiator to become an effective leader. Then the above conclusion can be interpreted differently: when the task is more difficult, fewer fish in the group have enough confidence to be an effective leader; however, more fish are confident when the task is easier.

### 4.3. Future work

In the future study, it is of great interest to explore the application of the proposed framework to investigate more complicated behavioural traits of robotic fish groups. One direction is to extend the robotic fish system to a heterogeneous system consisting of both robotic and real fish. Such a heterogeneous system may help biologists to understand better the interactive behaviours in real fish schools. By adding one or more robotic fish to a group of real fish, we might steer the behaviour of the robotic fish to influence the overall fish group. The evolving heterogeneous system may provide us an inspiring way of understanding fish behavioural traits in nature and designing new coordination strategies for autonomous robots.

## Supplementary Material

Supplementary figures

## Data accessibility

All data necessary to reproduce analyses have been uploaded as the electronic supplementary material.
