# Supplementary material for: Emergence of leadership in a robotic fish group under diverging individual personality traits
Source: R Soc Open Sci. 2017 May 24;4(5):161015. doi: 10.1098/rsos.161015 (PMC5451794; doi:10.1098/rsos.161015)
Supplement: Supplementary figures [file rsos161015supp1.pdf]

Emergence of leadership in a robotic fish group under diverging individual personality traits: Electronic Supplementary Material

Chen Wang[1], Xiaojie Chen[4], Guangming Xie[1,3], and Ming Cao[2]

[1]The State Key Laboratory of Turbulence and Complex Systems, Center for Systems and Control, College of Engineering, Peking University, 100871 Beijing, People's Republic of China
[2]Faculty of Science and Engineering, University of Groningen, Nijenborgh 4, 9747 AG Groningen, The Netherlands.
[3]Institute of Ocean Research, Peking University, 100871 Beijing, People's Republic of China
[4]School of Mathematical Sciences, University of Electronic Sciences and Technology of China, 611731 Chengdu, People's Republic of China

Correspondence and requests for materials should be addressed to
Chen Wang (email:wangchen@pku.edu.cn) and Ming Cao (email:m.cao@rug.nl)

SUPPLEMENTARY MATERIALS

Supplementary figures:

Methods

Figure S1. Software architecture of the experimental platform used to conduct our proposed framework to investigate the behaviors of biomimetic robotic fish groups. (Figure drawn by Chen Wang, Peking University.)

Figure S2. Relationship between the success rate and the number of the C-player (n_C) for different difficulty levels of the foraging tasks. The agent, who plays as an initiator or a follower and thus takes the action to cooperate to remove the obstacle in the foraging task, is called a cooperating fish (i.e., a C-player). The difficulty level increases from 1 to 4. (Figure drawn by Chen Wang, Peking University.)

Results

Figure S3. Typical evolutionary processes with fixed tasks of the most difficult level. (a)(b) correspond to the case when the update rate is small ($\lambda = 0.4$), for which the success rate is 56% and the diversity is 0.06; (c)(d) correspond to the case when the update rate is large ($\lambda = 0.9$), for which the success rate is 96% and the diversity is 0.37. (Figure drawn by Chen Wang, Peking University.)

Figure S4. Reference model. Each data point is the average of 20 runs and the standard error (shaded area) are shown as well. The x axis shows the difficulty level of the foraging task which increases from 1 to 4. (a) shows the number of cooperating fish (i.e., C-players) which takes the action to cooperate to remove the obstacle in the foraging task; (b) shows the success rate; (c) shows the average strategy; and (d) shows the average payoff. (Figure drawn by Chen Wang, Peking University.)

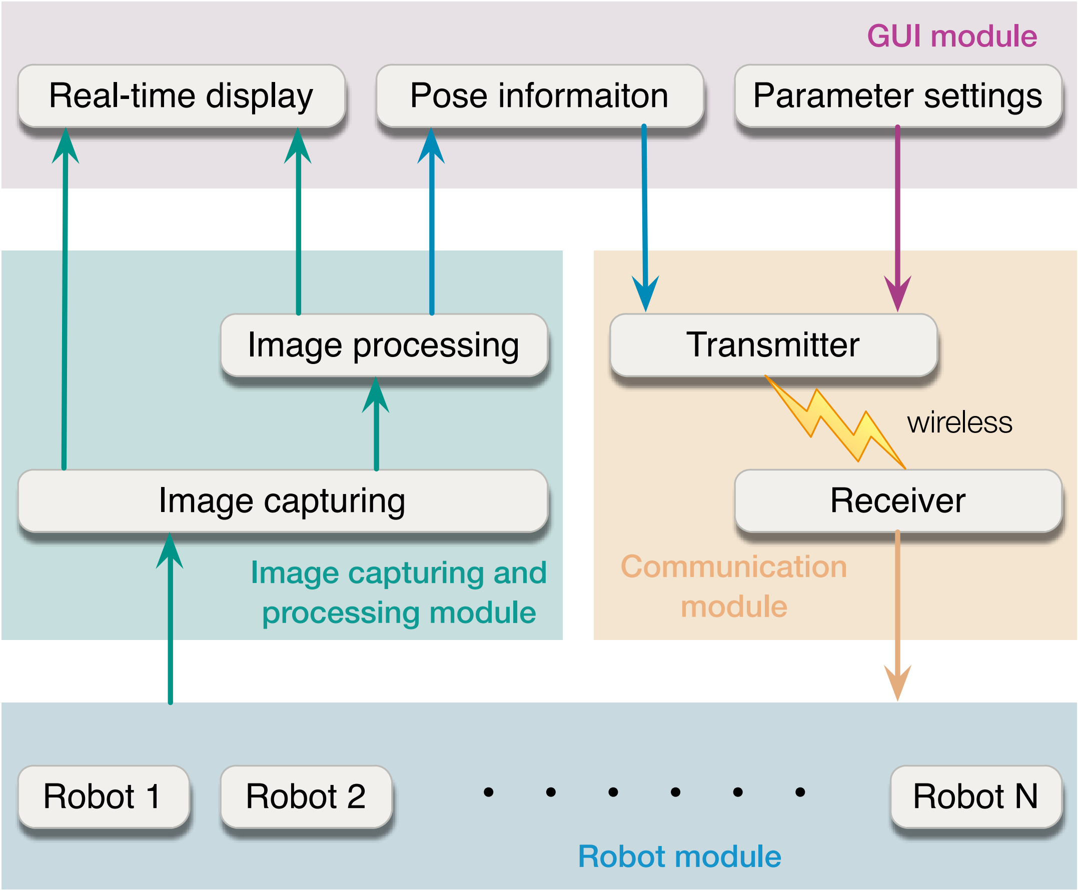

Supplementary Figure S1: Software architecture of the experimental platform used to conduct our proposed framework to investigate the behaviors of biomimetic robotic fish groups. (Figure drawn by Chen Wang, Peking University.)

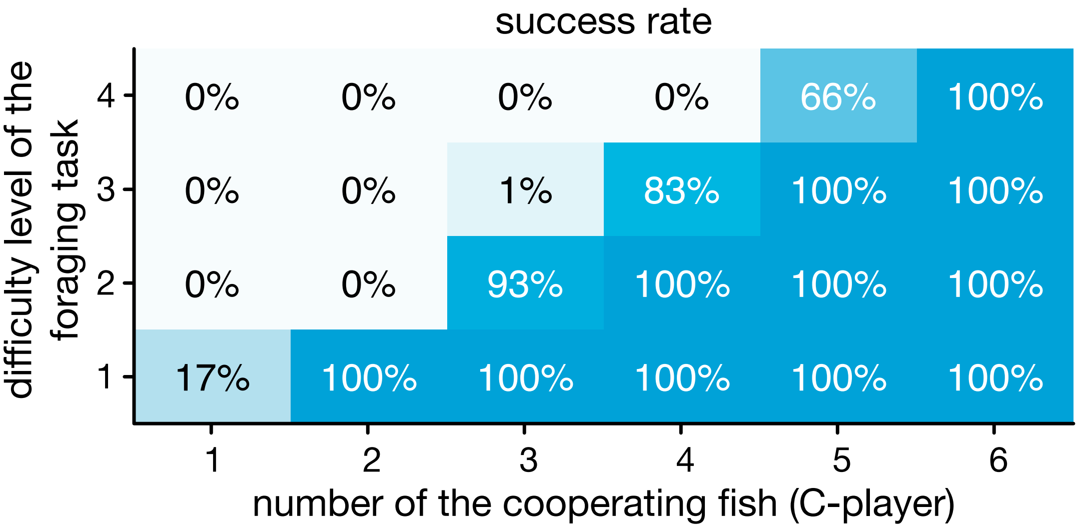

Supplementary Figure S2: Relationship between the success rate and the number of the C-player (n_C) for different difficulty levels of the foraging tasks. The agent, who plays as an initiator or a follower and thus takes the action to cooperate to remove the obstacle in the foraging task, is called a cooperating fish (i.e., a C-player). The difficulty level increases from 1 to 4. (Figure drawn by Chen Wang, Peking University.)

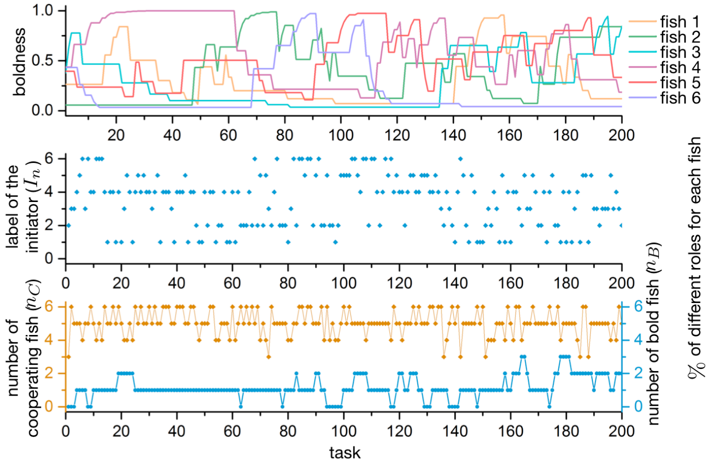

(a) $\lambda = 0.4$

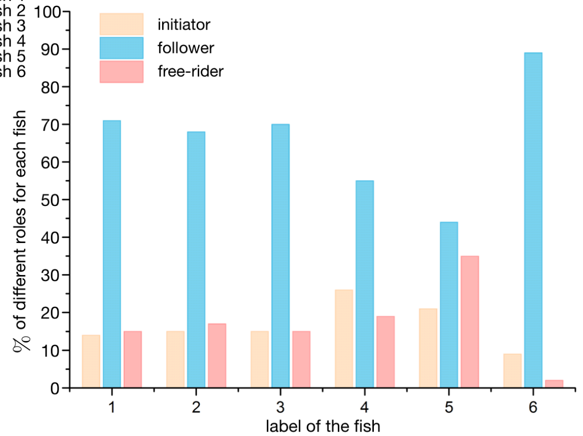

(b) $\lambda = 0.4$

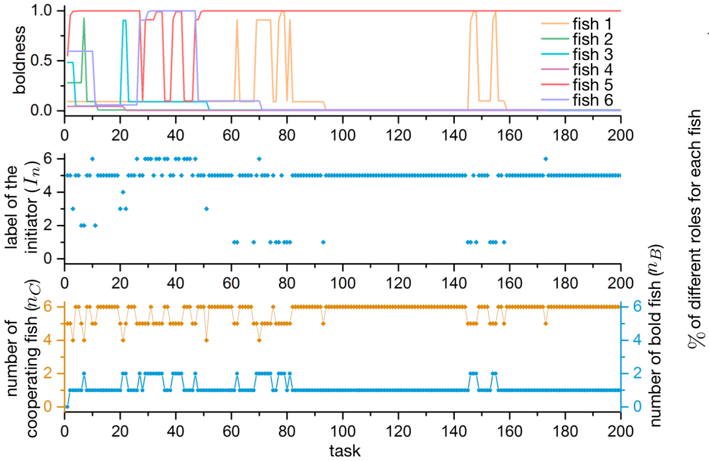

(c) $\lambda = 0.9$

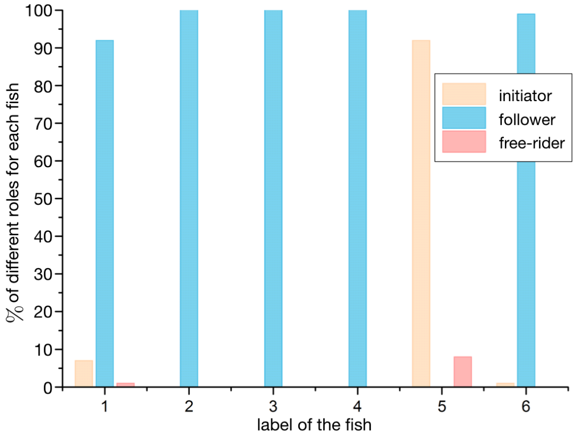

(d) $\lambda = 0.9$

Supplementary Figure S3: Typical evolutionary processes with fixed tasks of the most difficult level. (a)(b) correspond to the case when the update rate is small ($\lambda = 0.4$), for which the success rate is 56% and the diversity is 0.06; (c)(d) correspond to the case when the update rate is large ($\lambda = 0.9$), for which the success rate is 96% and the diversity is 0.37. (Figure drawn by Chen Wang, Peking University.)

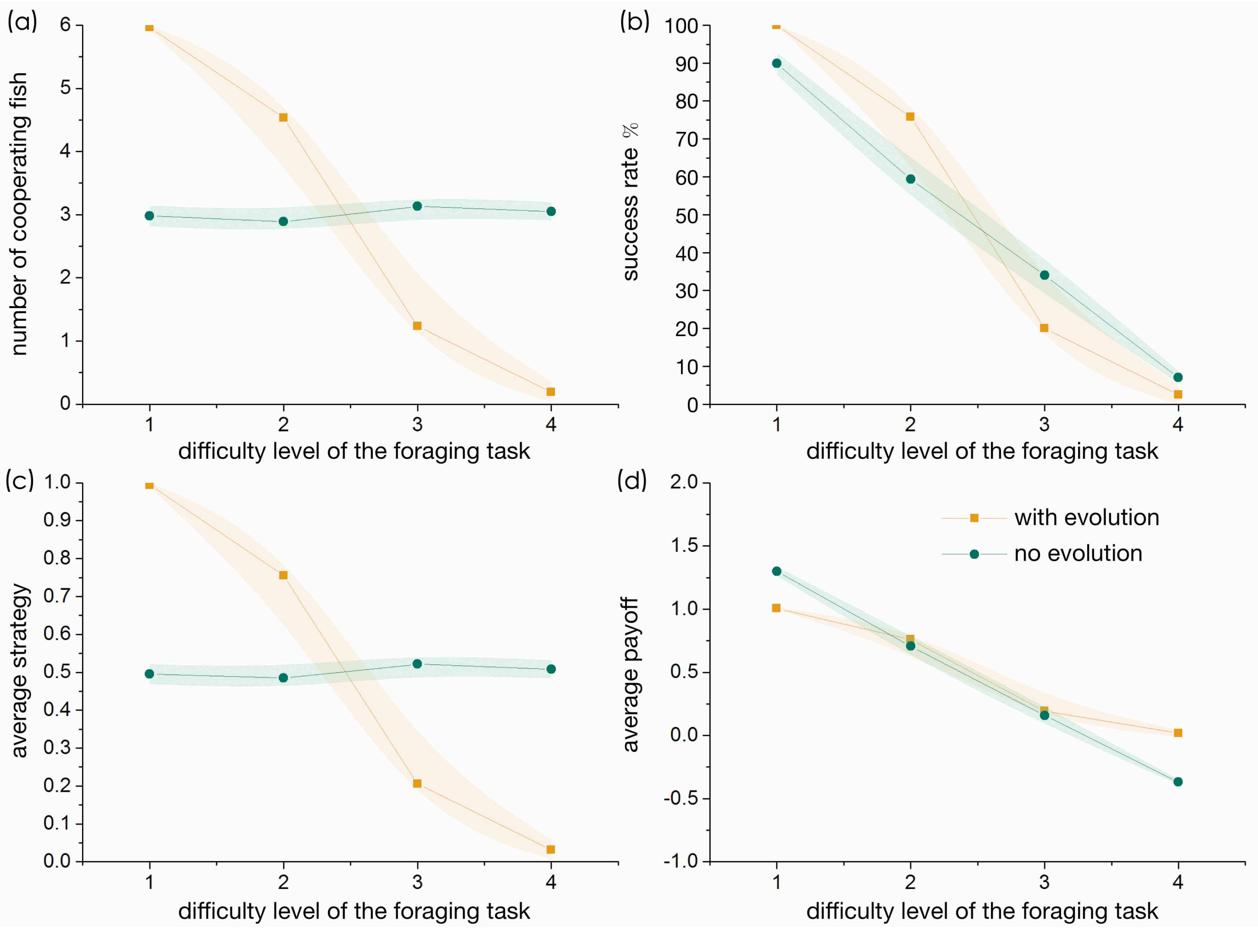

Supplementary Figure S4: Reference model. Each data point is the average of 20 runs and the standard error (shaded area) are shown as well. The x axis shows the difficulty level of the foraging task which increases from 1 to 4. (a) shows the number of cooperating fish (i.e., C-players) which takes the action to cooperate to remove the obstacle in the foraging task; (b) shows the success rate; (c) shows the average strategy; and (d) shows the average payoff. (Figure drawn by Chen Wang, Peking University.)
